# Spread of Carbapenemase-producing Enterobacteria in a Southwest Hospital in China

**DOI:** 10.1186/s12941-014-0042-4

**Published:** 2014-08-12

**Authors:** Sheng Chen, Wei Feng, Jianhong Chen, Wei Liao, Nianhai He, Qian Wang, Fengjun Sun, Peiyuan Xia

**Affiliations:** 1 Department of Pharmacy, Southwest Hospital, Third Military Medical University, Chongqing 400038, China; 2 Department of Pediatrics, Southwest Hospital, Third Military Medical University, Chongqing, China; 3 Department of Pharmacy, Xinqiao Hospital, Third Military Medical University, Chongqing, China

**Keywords:** Enterobacteria, Carbapenemase, Emergence, Spread, Resistance

## Abstract

**Background:**

The rapid emergence and dissemination of carbapenem resistance in Enterobacteriaceae complicates the treatment of infections caused by these organisms.

**Methods:**

We collected clinical isolates with meropenem inhibition zones of ≤ 22 mm from January 1, 2009, through December 31, 2010. We attempted to amplify the NDM-1 gene from these isolates and conducted the modified Hodge test (MHT). The minimal inhibitory concentration (MIC) of the MHT-positive strains was determined by the agar disk dilution method. The carbapenemase-encoding resistance genes of these strains were examined using polymerase chain reaction (PCR) analysis and a sequencing strategy to characterize these enzymes. The clonal relationship among isolates was analyzed by pulsed-field gel electrophoresis (PFGE).

**Results:**

Among the 158 Enterobacteriaceae isolates that were collected, there were no NDM-1-positive strains and 26 MHT-positive strains. Among the latter, 18 strains were IMP-4-positive, and 1 was KPC-2-positive. In addition, 15 of the IMP-4-positive *Klebsiella pneumoniae* strains belonged to 4 PFGE genotypes, with 8 strains having the same genotype.

**Conclusion:**

These results suggest that nosocomial infections are one of the main reasons for the spread of these resistant strains.

## Introduction

Enterobacteriaceae are the most common pathogenic bacteria. Recently, the emergence of resistant pathogenic bacteria has caused significant problems for the clinical treatment of enterobacterial infections worldwide. There are many mechanisms leading to this resistance [1]. Extended-spectrum β-lactamase (ESBL)-producing pathogenic enterobacteria is a serious problem for antibiotic management because the ESBL genes are easily transferred from one organism to another via plasmids or other mobile genetic elements in combination with non-β-lactam resistance mechanisms, leading to multidrug-resistant isolates [1],[2].

Carbapenem antibiotics are a class of β-lactam antibiotics that are often used as a last resort for treating enterobacterial resistance. However, an increasing number of studies are reporting carbapenem-resistant enterobacteria [2]-[4]. Carbapenem-resistant enterobacterial infections are now clearly associated with significant morbidity and mortality. However, few studies have monitored the spread of these Enterobacteriaceae. Here, we report the spread of IMP-4-producing enterobacteria in a hospital in Chongqing, China.

## Materials and methods

### Study design

The study period was from January 1, 2009, through December 31, 2010. All samples were collected from the Southwest Hospital, which is one of the largest teaching hospitals in Chongqing, China, with approximately 3000 beds. The bacterial isolates were identified in the clinical microbiology laboratory using standard biochemical tests and the Vitek system (BioMérieux, Hazelwood, MO). If the meropenem inhibition zone was ≤ 22 mm, an attempt was made to amplify the NDM-1 gene from the strain, and the strain was evaluated with the modified Hodge test (MHT) [5]. The MHT has been widely used to screen for carbapenemase activity because it directly analyzes this activity. If the sample was either MHT-positive or NDM-1-positive, the medical records and laboratory data for the associated patient were retrospectively reviewed using records available from the Southwest Hospital information system and from the microbiology laboratory. In this study, each isolate was collected from a different patient. This study was approved by the Ethics Committee of the Third Military Medical University (Approval number, KY200508).

### Susceptibility testing

Bacterial isolates were identified according to standard methods and tested against various antibiotics by the disk diffusion method; the zone diameters were interpreted according to the Clinical and Laboratory Standards Institute (CLSI) guidelines. The minimal inhibitory concentration (MIC) was evaluated for MHT-positive and NDM-1-positive strains. The MIC was determined by the agar disk dilution method in freshly prepared test medium (MH) according to the 2010 guidelines of the CLSI, and *Escherichia coli* strain ATCC 25922 was used as the control strain for the MIC testing. The resistance rate refers to the number of resistant strains divided by the total number of strains. The susceptible strains include those that are fully susceptible and those with an intermediate susceptible according to the CLSI standard.

### PFGE

All *Klebsiella pneumoniae* isolates were analyzed by pulsed-field gel electrophoresis (PFGE) using the contour-clamped homogeneous electric field (CHEF) technique, which is similar to the method described by Shen et al. [6] and Gouby et al. [7]. The XbaI restriction enzyme was used (Takara, DaLian, China). DNA fragments were separated by electrophoresis in a 1% agarose III gel (Bio-Rad, China) with a CHEF apparatus (CHEF Mapper XA, Bio-Rad). The electrophoresis was performed at 14°C and 6 V/cm and with alternating pulses at a 120° angle in a 2- to 40-s pulse-time gradient for 24 h. The typing criteria were based on the protocol described by Shen et al. [6] and Tenover et al. [8].

### Carbapenemase analysis

Carbapenemase genes that are common in China were amplified by PCR with the primers shown in Table 1. NDM-1-, IMI-1-, SPM-1-, IMP-1-, KPC-1-, VIM-1-, and OXA-type-positive strains were preserved in our laboratory [9]. The sequence analyses were performed using the BLAST program available on the National Center for Biotechnology Information server (http://www.ncbi.nlm.nih.gov/).

**Table 1:** The primers used to amplify the carbapenemase genes by PCR

**Gene**	**Primer**	**Size (bp)**	**Reference**
IMP-1 F	5‘-atgagcaagttatctgtattctttat-3’	741	9
IMP-1R	5‘-ttagttgcttagttttgatggttt-3’		
KPC-1 F	5‘-tcgccgtctagttctgctgtctt-3’	965	9
KPC-1R	5‘-ccgcgcagactcctagcctaa-3’		
NDM-1 F	5‘-tcaccgagattgccgagcga-3’	457	9
NDM-1R	5‘-gggcagtcgcttccaacggt-3’		
VIM-1 F	5‘- ggtcgcatatcgcaacgcagt-3’	636	9
VIM-1R	5‘-cggcgactgagcgatttttg-3’		
IMI-1 F	5‘- ccattcacccatcacaac-3’	440	9
IMI-1R	5‘- ctaccgcataatcatttgc-3’		
SPM-1 F	5‘- ctgcttggattcatgggcgc-3’	783	1
SPM-1R	5‘- ccttttccgcgaccttgatc-3’		
OXA-23 F	5‘- acttgctatgtggttgcttctctt-3’	797	9
OXA-23R	5‘- ttcagctgttttaatgatttcatca-3’		
OXA-24 F	5‘- cgatcagaatgttcaagcgc-3’	559	9
OXA-24R	5‘- acgattctcccctctgcgc-3’		
OXA-48 F	5‘- ttggtggcatcgattatcgg-3’	744	1
OXA-48R	5‘- gagcacttcttttgtgatggc-3’		
OXA-51 F	5‘- tccaaatcacagcgcttcaaaa-3’	639	9
OXA-51R	5‘- tgaggctgaacaacccatcca-3’		
OXA-58 F	5‘- cgatcagaatgttcaagcgc-3’	529	1
OXA-58R	5‘- acgattctcccctctgcgc-3’		

## Results

From January 1, 2009 to December 31, 2010, 158 Enterobacteriaceae isolates (92 *K. pneumoniae*, 9 *E. coli*, and 57 *Enterobacter cloacae*) with reduced susceptibility to imipenem were identified in the hospital. Only 1 isolate of each bacterial species was selected per patient, and all of the strains were collected from patients 48 h after admission. Among the 158 Enterobacteriaceae, the Hodge test was positive for 26 strains (20 *K. pneumoniae*, 4 *E. coli*, and 2 *E. cloacae*), and no strain was NDM-1-positive. The features of these isolates and the patients are presented in Table 2. Among twenty-six clinical strains, eighteen isolates were IMP-4 positive, and one was KPC-2 positive. Seventeen strains were from sputum, and sixteen strains were from pediatrics. All the strains of group A PFGE genotype were from pediatrics.

**Table 2:** **Features of the** ***Enterobacteriaceae*** **clinical isolates**

	**Collection date**	**Strain**	**Gender**	**Age**	**Specimen**	**Hospital days**	**Ward**	**Diagnosis**	**PFGE genotype**	**Carbapenemase gene**	**IMP-MIC**	**MEM-MIC**
1	2009-9-28	Kpn	Male	31 days	Sputum	11	Pediatrics	Acute pneumonia	A	IMP-4	2	1
2	2009-10-7	Kpn	Female	2 days	Blood	7	Pediatrics	Neonatal pneumonia	A	IMP-4	4	2
3	2009-12-19	Kpn	Female	10 days	Sputum	6	Pediatrics	Neonatal pneumonia	A	IMP-4	<1	<1
4	2009-12-26	Kpn	Male	56 years	Abdominal fluid	31	Hepatobiliary department	Abdominal infection	C	-	8	8
5	2010-4-3	Kpn	Male	30 days	Sputum	8	Pediatrics	Acute pneumonia	E	IMP-4	64	128
6	2010-4-10	Eco	Female	57 years	Abdominal fluid	21	ICU	Severe acute pancreatitis	-	IMP-4	4	2
7	2010-5-15	Ecl	Male	15 days	Sputum	22	Pediatrics	Neonatal pneumonia	-	IMP-4	4	8
8	2010-5-24	Kpn	Male	58 minutes	Sputum	11	Pediatrics	Neonatal pneumonia	A	IMP-4	4	4
9	2010-5-26	Kpn	Male	38 minutes	Sputum	11	Pediatrics	Neonatal pneumonia	E	IMP-4	16	16
10	2010-5-29	Kpn	Male	2 months	Sputum	21	Pediatrics	Acute pneumonia	B	IMP-4	128	64
11	2010-5-29	Kpn	Male	6 hours	Sputum	12	Pediatrics	Neonatal pneumonia	A	IMP-4	4	4
12	2010-6-3	Kpn	Female	50 minutes	Sputum	88	Pediatrics	Neonatal pneumonia	A	-	4	2
13	2010-6-29	Kpn	Female	19 days	Sputum	12	Pediatrics	Neonatal pneumonia	A	IMP-4	<1	<1
14	2010-6-29	Eco	Male	56 years	Ascites	37	Department of hepatobiliary	Carcinoma of the pancreatic head	-	IMP-4	<1	<1
15	2010-7-3	Kpn	Male	20 years	Sputum	49	ICU	Open craniocerebral injury	E	-	4	4
16	2010-7-3	Eco	Male	79 years	Sputum	6	Department of gastroenterology	Chordapsus, pneumonia	-	-	<1	<1
17	2010-7-17	Kpn	Male	1 hour	Sputum	9	Pediatrics	Neonatal pneumonia	A	IMP-4	1	<1
18	2010-7-21	Kpn	Male	1 hour	Sputum	7	Pediatrics	Neonatal pneumonia	A	-	2	1
19	2010-7-21	Eco	Female	44 years	Blood	46	Department of hepatobiliary	Congenital choledochus cyst	-	-	2	1
20	2010-7-23	Kpn	Female	16 days	Sputum	10	Pediatrics	Neonatal pneumonia	E	IMP-4	32	16
21	2010-7-23	Kpn	Male	1 hour	Sputum	18	Pediatrics	Neonatal pneumonia	C	IMP-4	32	32
22	2010-7-23	Kpn	Male	60 years	Blood	46	ICU	Multiple injuries	E	IMP-4	8	8
23	2010-10-11	Kpn	Female	26 days	Sputum	12	Pediatrics	Neonatal pneumonia	A	IMP-4	2	2
24	2010-10-17	Ecl	Male	54 years	Wound	48	ICU	Penetrating wound	-	-	1	<1
25	2010-10-20	Kpn	Female	69 years	Wound	17	Department of neurology	Pneumonia	D	KPC-2	16	8
26	2010-10-20	Kpn	Female	64 years	Wound	34	Burn	Burn	B	IMP-4	2	2

The results of MIC range, MIC_50_ and MIC_90_ analyses are presented in Table 3. We used previously described PCR reactions to screen for carbapenem-hydrolyzing β-lactamase genes. The PCR reactions for the IMP-4 and KPC-2 genes were positive [9]. The major epidemic pattern A consisted of ten IMP-4-positive isolates from the Department of Pediatrics. However, the only one KPC-2-positive strain was collected from the Department of Neurology.

**Table 3:** **MIC range, MIC**_**50**_**and MIC**_**90**_**for antibiotics tested against Enterobacteriaceae**

**Antimicrobial agent**	**MIC range (μg/ml)**	**MIC**_**50**_**(μg/ml)**	**MIC**_**90**_**(μg/ml)**
Imipenem	1 < − 128	4	16
Meropenem	1 < − 64	2	16
Ceftazidim	1 < − > 512	512	> 512
Ceftriaxone	1 < − > 512	> 512	> 512
Piperacillin/tazobactam	1 < − > 512	128	512
Piperacillin	1 − > 512	> 512	> 512
Ciprofloxacin	1 < − 64	4	32
Levofloxacin	1 < −128	8	32
Gentamicin	1 < − >512	8	64
Tobramycin	1 < − >512	4	64

**Figure 1:**
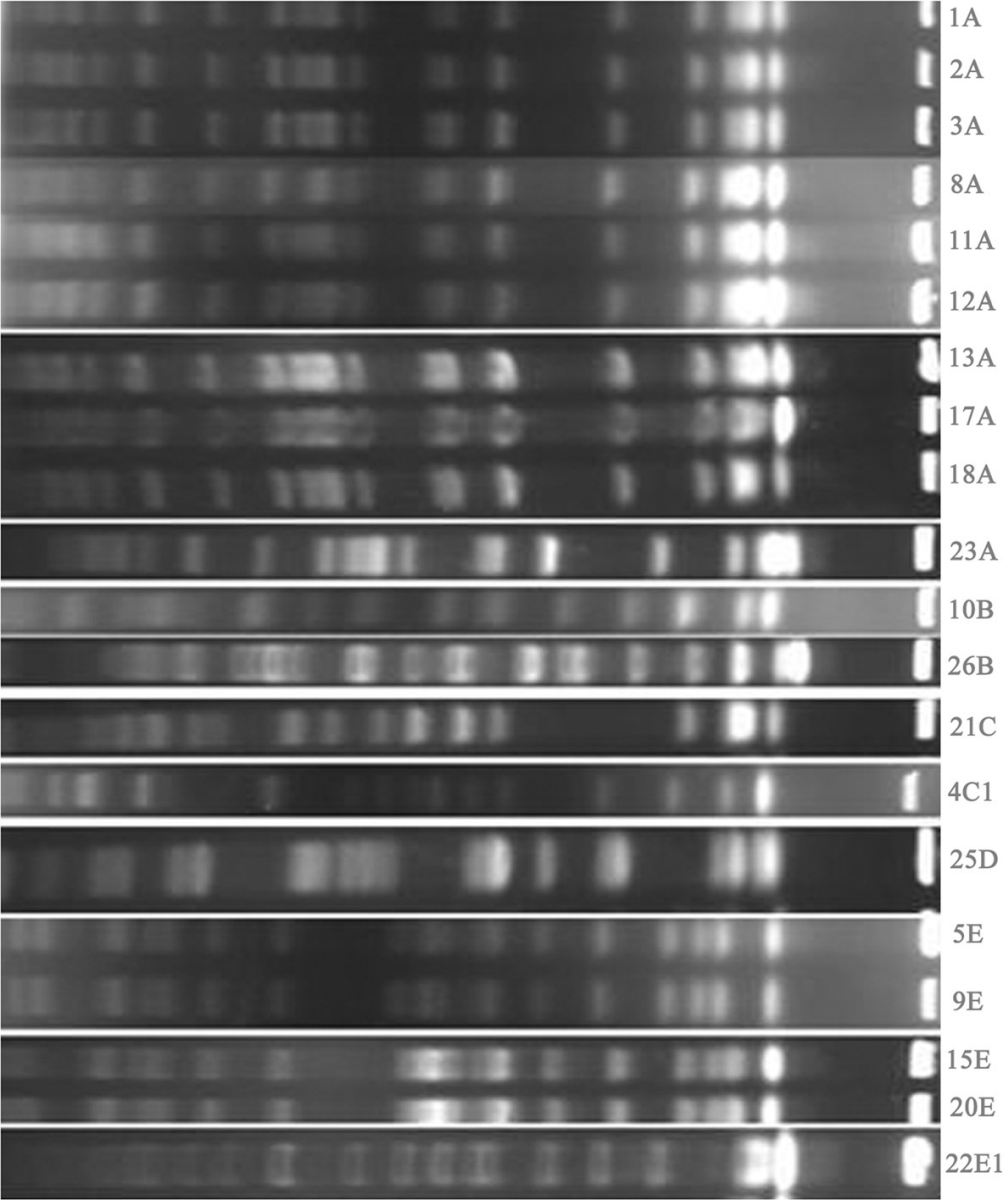
**PFGE analysis of genomic DNA from isolates of** ***K. pneumoniae.***

XbaI pulsed-field gel electrophoresis was then used to study the genetic relatedness of the 20 *K. pneumoniae* isolates. The results are presented in Figure 1, and they suggest nosocomial transmission.

## Discussion

In the present study, we analyzed the carbapenemase genes and assessed the spread of IMP-4-positive enterobacteria in a hospital in Chongqing, China. IMP-4 is a common MBL and was first reported in *Acinetobacter baumannii* in Hong Kong, China; it subsequently spread throughout the world, particularly to mainland China and Australia [10],[11]. IMP-4 has been reported in enterobacteria in China [12] but has not been found in Southwestern China. Previously, we found that the IMP-4 gene existed in *A. baumannii* (data were not shown) and *Pseudomonas aeruginosa*[9]. Since 2007, we have detected the carbapenemase genes in enterobacteria. In 2009, we found the first carbapenemase gene, IMP-4, which we subsequently showed to be spreading among enterobacteria. However, IMP-8 has been reported in Chongqing despite the presence of IMP-4 [13]. These results suggested that IMP-4 may be transferred to enterobacteria from other bacteria in our hospital. After the first IMP-4-positive *K. pneumoniae* strain was found in the Pediatrics Unit, 18 IMP-4-positive enterobacteria were identified, including one *E. cloacae and two E. coli* strains. KPC-2 is common in the coastal cities of China, such as Hangzhou and Shanghai [14],[15]. Only one KPC-2-positive strain was found among the clinical isolates, and it was reported in The First Affiliated Hospital of Chongqing Medical University, Chongqing [13]. Since then, we have not found another KPC-2-positive strain. Thus, the KPC-2-positive strain may have been present in the patient prior to hospitalization and not acquired from a hospital ward.

For seven MHT-positive strains, no carbapenemase gene was amplified. The MHT has negative and positive likelihood rates, and the positive likelihood rate in our experiment is higher than that in a previous report [16]. Here, we only tested for the commonly appearing carbapenemase genes in China, so it is possible that these seven strains have other resistance genes. The MHT- and IMP-4-positive strains exhibited low-level resistance and even susceptibility to carbapenems in our study, and Nordmann reported similar results [2]. Thus, the highly resistant strains carrying IMP-4 must have additional mechanisms for carbapenem resistance, such as outer-membrane permeability defects, other resistance genes, or target alteration. Therefore, to treat carbapenemase-positive strains, carbapenem antibiotics, although not the preferred choice, may be the only choice.

The PFGE revealed genetic diversity among the 20 *K. pneumoniae* isolates, which belonged to five PFGE types. Ten strains of *K. pneumoniae* shared the same PFGE type. The phenomenon of the same clones spreading within the same wards or throughout different wards during the same period was observed. The same clones had similar resistance results but slightly different MIC results, suggesting that some features of the bacteria had changed as they spread and replicated or that these bacteria could easily exchange mobile genetic elements. These findings indicated that the decreased susceptibility to carbapenems in *K. pneumoniae* may arise by the stepwise accumulation of multiple carbapenem resistance determinants in different clones. These results suggest that this type of infection can be classified as a hospital-derived infection. Therefore, controlling hospital infections is a method for reducing the accumulation of bacteria resistance determinants.

The average hospital stay of the patients with these isolates was 23.1 days, while the average hospital stay of all patients in our hospital was 9.8 days. Long-term acute care hospitals (LTACHs) may play a particularly important role in the spread of KPC-producing Enterobacteriaceae, particularly among neonates or patients with a serious illness who have a reduced immune system functional capacity and who are undergoing invasive examinations or treatments. Our results support the conclusions reported by Endimiani [17]. Thus, for patients with a high risk of infection, reinforcement of infection control measures based on the guidance for carbapenemase-producing Enterobacteriaceae prevention [17] with early warning is particularly critical and should include promotion of hand hygiene. In addition, the immediate initiation of contact precautions is necessary, which can lead to a considerable reduction in hospital-acquired carbapenemase producers. Our findings also suggest that a significant containment of carbapenem-resistant *K. pneumoniae* strains from the environment is possible with the implementation of a comprehensive infection control intervention program that includes active surveillance, carrier isolation/cohorting, and a dedicated staff to treat individuals who become infected. The advent of these tests has enabled the early detection of clonal strains that are likely to be introduced into our hospital, especially from patients with previous hospitalizations. In the future, we will analyze more diverse strains and resistant genes. After infection control measures are reinforced, we will conduct additional infection control studies to test the effects of reinforcing infection control measures.

Taken together, these findings reveal the process involved in the recent spread of IMP-4 in our hospital. Carbapenem-resistant enterobacteria have become a significant concern in our hospital. The presence of carbapenemases, such as KPC-2 and IMP-4, may have played a significant role in the development of these resistant strains. Cooperation among the clinical microbiology laboratory, the infection control team, and the medical and nursing staff is vital for the design and implementation of appropriate infection control measures for carbapenemase-producing pathogens. The use of active surveillance as part of this multifactorial intervention may also aid in decreasing the secondary transmission rates of imported genes. Other interventions, such as shortening the average length of hospital stays, may further augment these infection control actions. Examining whether our interventions change the prevalence of other, less common, multidrug-resistant pathogens will also be of interest.

## Competing interests

The authors declare that they have no competing interests.

## Authors’ contributions

SC carried out the protocol design and drafted this manuscript. WF carried out the collection and analysis of data for this study, and revised the content. JC carried out the collection of data, the analysis and interpretation of these data. WL, NH and QW participated in the collection of data for this study. FS and PX carried out the protocol design, collection of data, the analysis and interpretation of these data, and drafted and revised the content of this manuscript. All authors read and approved the final manuscript.
